# The Translation Regulatory Subunit eIF3f Controls the Kinase-Dependent mTOR Signaling Required for Muscle Differentiation and Hypertrophy in Mouse

**DOI:** 10.1371/journal.pone.0008994

**Published:** 2010-02-01

**Authors:** Alfredo Csibi, Karen Cornille, Marie-Pierre Leibovitch, Anne Poupon, Lionel A. Tintignac, Anthony M. J. Sanchez, Serge A. Leibovitch

**Affiliations:** 1 Laboratoire de Génomique Fonctionnelle et Myogenèse, UMR866 Différenciation Cellulaire et Croissance, SupAgro-INRA, Montpellier, France; 2 Biologie et Bioinformatique des Systèmes de Signalisation, UMR Physiologie de la Reproduction et des Comportements, INRA, Nouzilly, France; 3 Equipe Remodelage Musculaire et Signalisation, UMR866 Différenciation Cellulaire et Croissance, SupAgro-INRA, Montpellier, France; Roswell Park Cancer Institute, United States of America

## Abstract

The mTORC1 pathway is required for both the terminal muscle differentiation and hypertrophy by controlling the mammalian translational machinery via phosphorylation of S6K1 and 4E-BP1. mTOR and S6K1 are connected by interacting with the eIF3 initiation complex. The regulatory subunit eIF3f plays a major role in muscle hypertrophy and is a key target that accounts for MAFbx function during atrophy. Here we present evidence that in MAFbx-induced atrophy the degradation of eIF3f suppresses S6K1 activation by mTOR, whereas an eIF3f mutant insensitive to MAFbx polyubiquitination maintained persistent phosphorylation of S6K1 and rpS6. During terminal muscle differentiation a conserved TOS motif in eIF3f connects mTOR/raptor complex, which phosphorylates S6K1 and regulates downstream effectors of mTOR and Cap-dependent translation initiation. Thus eIF3f plays a major role for proper activity of mTORC1 to regulate skeletal muscle size.

## Introduction

The mammalian target of rapamycin (mTOR, also known as FRAP, RAFT1 or RAPT) has emerged as a critical nutritional and cellular energy checkpoint sensor and a regulator of cell growth [1]–[3] This evolutionary conserved Ser/Thr kinase is a member of the PIKK family of protein kinases [2] controlling many cellular processes, including protein synthesis, ribosome biogenesis, nutrient transport and autophagy [4]. mTOR assembles in two distinct multiprotein complexes, termed mTORC1 and mTORC2 [5], [6]. mTORC1 consists of raptor (regulatory associated protein of mTOR), mLST8, PRAS40 and mTOR [7] and is sensitive to rapamycin. mTORC2 consists of rictor (rapamycin insensitive companion of mTOR), mSIN1, mLST8 and mTOR [5], [6]. In response to growth factors, hormones and amino acids, mTORC1 is classically known to regulate cell growth and proliferation through modulation of protein synthesis by phosphorylation toward its downstream effectors, S6K1 [8] and 4E-BP1 [1]. Phosphorylation of 4E-BP1 promotes its dissociation from eIF4E bound to the mRNA 7-methylguanosine cap structure, allowing the assembly of the preinitiation complex (PIC), composed of eIF3, eIF4F, 40S ribosomal subunit and the ternary complex eIF2/GTP/Met-tRNA [9]. S6K1 activation needs initial phosphorylation by mTORC1 on T389 [10] and additional inputs on T229 for fully activation by the phosphoinositide-dependent kinase 1 (PDK1) [11]. S6K1-mediated regulation of translation is thought to occur through phosphorylation of the 40S ribosomal protein S6. Thus, the increased activation of S6 is linked to cellular growth control [12].

Changes in the size of adult muscle, in response to external stimuli, are mainly due to the growth of individual muscle fibers rather than an increase in fiber number [13]. Muscle hypertrophy is associated with increased protein synthesis [14]. Previous studies pointed towards a key role of mTOR as a regulator of skeletal muscle growth *in vivo* and *in cellulo*. For example, rapamycin inhibits recovery of skeletal muscle from atrophy [15]. Moreover, activation of Akt/PKB (upstream regulator of mTORC1) induces muscle hypertrophy in a rapamycin-sensitive fashion [15]–[17]. In contrast, muscle fibers of mice deficient for S6K1 are atrophic [18] and muscle-specific ablation of raptor prevents the phosphorylation of 4E-BP1 and S6K1 and results in muscle dystrophy [19]. However, it is not yet clear how mTOR and S6K1 regulate the translational machinery in skeletal muscle.

The regulatory subunit of the eIF3 (eukaryotic Initiation Factor 3) complex; eIF3f, is a member of the Mov34 family, containing an Mpr1/Pad central motif [20]. eIF3f function within the eIF3 complex remains to be defined. However, it is essential for *Schizosaccharomyces pombe* viability, and its depletion markedly decreased the global protein synthesis in fission yeast [21]. eIF3f overexpression has been associated with inhibition of HIV-1 replication [22] and with activation of apoptosis in melanoma and pancreatic cancer cells [23]. In skeletal muscle, eIF3f has been reported as a crucial checkpoint in the crossroads of signaling pathways controlling muscle size [24]. On one hand, eIF3f has been identified as a major target that accounts for MAFbx/Atrogin-1 function during skeletal muscle atrophy [25] and could explain that muscle atrophy involves a suppression of the same program of gene expression that is activated during work-induced hypertrophy or by IGF in normal growth [26]. On the other hand, previous studies have identified eIF3 complex as a scaffold for the rate-limiting step in protein translation, the association of mTOR and S6K1 (among other components) leading to the formation of the PIC [27], [28]. These findings suggest an important role of this initiation factor in the process of protein synthesis in skeletal muscle. Indeed, overexpression of eIF3f in muscle cells and in adult skeletal muscle induces hypertrophy associated with an increase of sarcomeric proteins. In contrast, eIF3f repression in differentiated skeletal muscle induces atrophy [25]. Furthermore, an eIF3f mutant insensitive to MAFbx polyubiquitination (eIF3f K_5–10_R) shows enhanced hypertrophic activities *in vivo* and *in cellulo* [29].

However, little is known about the mechanistic underlying the eIF3f-mediated hypertrophy and its relation with mTOR and S6K1 in skeletal muscle.

In the present work, we show in MAFbx-induced atrophy that the decreased activity of mTORC1 is correlated with the degradation of eIF3f and inversely mTOR and its downstream targets S6K1 and 4E-BP1 via eIF3f control muscle size. mTOR and S6K1 physically interact with two different domains of eIF3f. The Mov34 motif, involved in MAFbx-mediated polyubiquitination of eIF3f [25] is responsible for the interaction with the inactive hypophosphorylated form of S6K1. mTOR interacts with the C-terminal domain of eIF3f, a region recently shown to be critical for proper eIF3f activity in skeletal muscle [29]. Moreover, an increase in both affinity and accessibility of mTOR and raptor for the conserved TOS motif present in the COOH domain of eIF3f elucidates the hypertrophic activity of the mutant eIF3f K_5–10_R. In muscle hypertrophy eIF3f not only up-regulates the muscle structural proteins expression but also increases the association of translational components with the 7-methylguanosine-cap complex and activates Cap-dependent translation. These different observations lead us to envision the involvement of the eIF3f regulatory subunit acting as a scaffold to coordinate mTOR and S6K1 mediated the assembly of a PIC specific to mRNAs encoding proteins involved in muscle hypertrophy.

## Results

### Degradation of eIF3f by MAFbx Suppresses S6K1 Activation by mTOR

**Figure 1:**
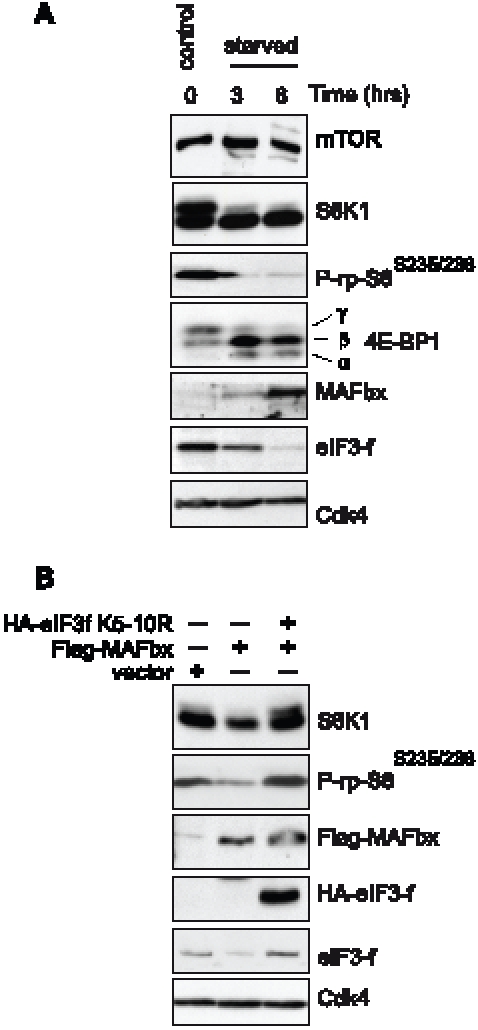
regulation of mTORC1 activity is correlated to MAFbx mediated eIF3f degradation during skeletal muscle atrophy. (**A**) Effects of starvation on components of the mTOR/S6K1 kinase pathway. Mouse primary cultured satellite cells myotubes at 4^th^ day of differentiation were starved by removal of growth medium and incubated in PBS for the indicated times. Proteins were extracted and subjected to immunoblots analysis. (**B**) Primary cultures of satellite cells were transfected with expression vectors encoding Flag-tagged MAFbx and/or the HA-tagged mutant eIF3f K_5–10_R and cultured in differentiation medium for 4 days. Total cellular lysates were analyzed by immunoblotting.

Food deprivation leads to rapid muscle wasting and increase in MAFbx expression [30], [31]. This increase of atrogin-1/MAFbx is related to polyubiquitination of eIF3f for further proteasome-mediated degradation [25]. Furthermore, food deprivation is accompanied with decreased phosphorylation of intermediate proteins of the PI3K/Akt/mTOR pathway although mTOR phosphorylation does not change during myotubes atrophy [32]. To address the question of whether the loss of eIF3f during muscle atrophy is implicated with the observed down regulation of mTOR activity, we assessed the phosphorylation of downstream targets of mTORC1 in starving mouse primary myotubes. After 6hr of serum and nutrients deprivation, MAFbx levels were increased, leading to a decrease of eIF3f. As expected, mTOR protein levels did not change. In contrast, atrophy induced a dramatic reduction of phosphorylation of 4E-BP1, S6K1 and its target the rpS6 (Figure 1A). On the other hand, overexpression of an eIF3f mutant protein lacking the MAFbx polyubiquitination sites (eIF3f K_5–10_R) [29] is associated with persistent phosphorylation of these mTOR downstream targets during starvation-induced atrophy, suggesting the implication of eIF3f degradation on the decreased activity of mTORC1 signaling during muscle atrophy. To confirm this, expression vectors coding for Flag-MAFbx and HA-eIF3f mutant K_5–10_R were co-transfected into mouse primary muscle cells. Overexpression of MAFbx led to myotubes atrophy [33] and a reduction of both the levels of endogenous eIF3f and the phosphorylation of S6K1. In contrast, overexpression of the stable mutant eIF3f K_5–10_R in the presence of MAFbx was associated with higher phosphorylation of S6K1 and protection of endogenous eIF3f (Figure 1B). Thus, these data suggest that during muscle atrophy the decreased activity of mTORC1 is correlated with the degradation of eIF3f and the accumulation of unphosphorylated forms of S6K1.

### mTOR and S6K1 Physically Interact with Two Different Domains of eIF3f

**Figure 2:**
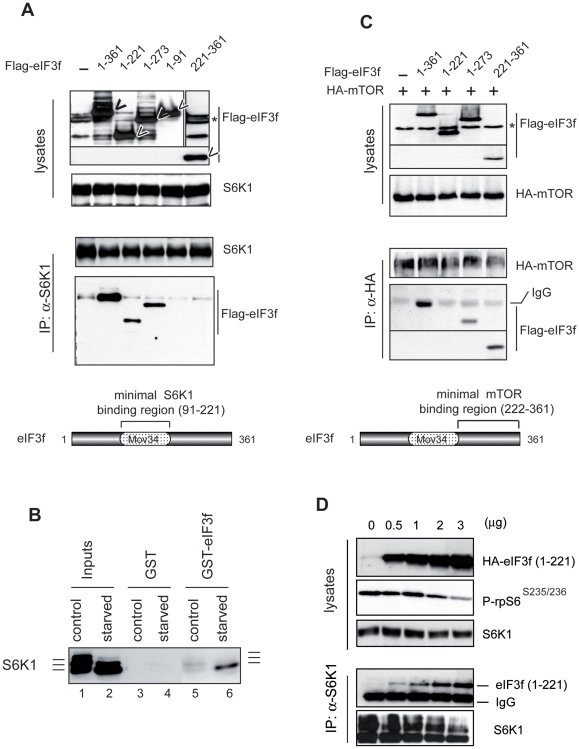
mTOR and S6K1 physically interact with two different domains of eIF3f. (**A**) Interaction of eIF3f mutants with S6K1. Mouse primary cultured satellite cells were transfected with expression vectors encoding Flag-tagged eIF3f wt and deletion mutants of eIF3f. Total cellular extracts were subjected to immunoprecipitation with anti-S6K1 followed by immunoblotting analysis with anti-Flag antibodies. (**B**) Phosphorylation of S6K1 regulates the interaction with eIF3f. Interaction of hyperphosphorylated (control) or hypophosphorylated S6K1 (starved) was tested for binding to eIF3-f. GST or GST-eIF3f beads were incubated with total cellular extracts (300 µg) of mouse primary culture of satellite myotubes in differentiation medium (control) or starved for 3h. Bound proteins were eluted, subjected to SDS-PAGE and analyzed by immunoblotting. (**C**) Interaction of eIF3f mutants with mTOR. Same as in (B) except that mouse primary cultured satellite cells were cotransfected with expression vector encoding HA-tagged mTOR. (**D**) An eIF3f mutant deleted of the mTOR-binding domain suppresses S6K1 phosphorylation. Mouse primary cultured satellite cells were transfected with increasing amounts of expression vectors encoding the deletion mutants eIF3f (aa1–221). Total cellular extracts were subjected to immunoprecipitation with anti-S6K1 followed by immunoblotting.

Recent data proved that the eIF3 complex acts as a scaffold to coordinate mTOR and S6K1 mediated translation [27]. However, the correlation between mTOR and S6K1 activation and their respective association and dissociation from eIF3 complex need still to be determined. The results outlined above and thus previously described [27], [28] suggested that in muscle cells the activation state of S6K1 could be governed by its binding to eIF3f. Thus we tested a panel of deletion mutants of eIF3f for their ability to coimmunoprecipitate with endogenous S6K1. As shown in Figure 2A, we found that the central domain containing amino acid residues 91–221 is sufficient for a high specific binding to S6K1. The protein sequence of this domain was found to correspond to the Mov34 domain of eIF3f. Because the phosphorylation of the hydrophobic motif at T389 in S6K1 has been shown to regulate the interaction between S6K1 and the eIF3-PIC complex [27], this prompted us to examine the phosphorylation state of S6K1 that physically interacts with eIF3f by using a GST pull down assay with total cellular extracts from normal myotubes and/or atrophied myotubes. As shown in Figure 2B, we observed that the hypophosphorylated forms of S6K1 preferentially bound to eIF3f. Our data are in agreement with the previous observations that S6K1 phosphorylation corresponding to its activation, promotes its dissociation from eIF3f.

To map the domain of eIF3f that interacts with mTOR, the same panel of deletion mutants of eIF3f was cotransfected with an expression vector encoding HA-tagged mTOR in mouse primary muscle cells. Total cell lysates were immunoprecipitated with anti-HA antibodies and eIF3f mutants were detected by immunoblotting with anti-Flag antibodies. We found that the C-terminal domain of eIF3f mediated its binding to mTOR (Figure 2C). To further confirm that eIF3f mediates activation of S6K1 by mTOR, an eIF3f mutant deleted of the C-terminal domain was transfected in mouse primary muscle cells and the phosphorylation of downstream targets of mTOR was assessed. As shown in Figure 2D, increasing amounts of the mutant (eIF3f 1–221) disrupted the activation of S6K1 by mTOR as evidenced by the decrease in both the S6K1 and rpS6 phosphorylation and an increase in hypophosphorylated form of S6K1 bound to eIF3f. Altogether our data confirm that eIF3f connects mTOR kinase to S6K1 and the non-phosphorylated forms of S6K1 physically interact with eIF3f. Interestingly, we previously reported that the C-terminal domain of eIF3f is believed to be critical for proper regulation and contribute to a fine-tuning mechanism that plays an important role for eIF3f function in skeletal muscle [29].

### mTOR and Its Downstream Targets Control Muscle Size via eIF3f

Recent data established mTOR as an important element in skeletal myocyte differentiation and hypertrophy. mTOR seems to promote the initial myoblast-myoblast fusion via a kinase-independent function involving the action of PLD1 and IGF-II [34], [35]. However, in late differentiation, the kinase activity of mTOR becomes necessary for the formation of mature myotubes, by modulating the activity of 4E-BP1 and S6K1 [34]. The latter has been shown to be essential for the control of muscle cytoplasmic volume [18]. Among the known substrates of S6K1, the ribosomal protein S6 (rpS6) seems to be directly involved in the control of cell size [12]. Given that eIF3f accumulates during skeletal muscle differentiation and that its genetic activation causes hypertrophy while its repression induces atrophy [25], we hypothesized that eIF3f plays a major role in mediating the mTOR-dependent control of late myogenesis and hypertrophy.

**Figure 3:**
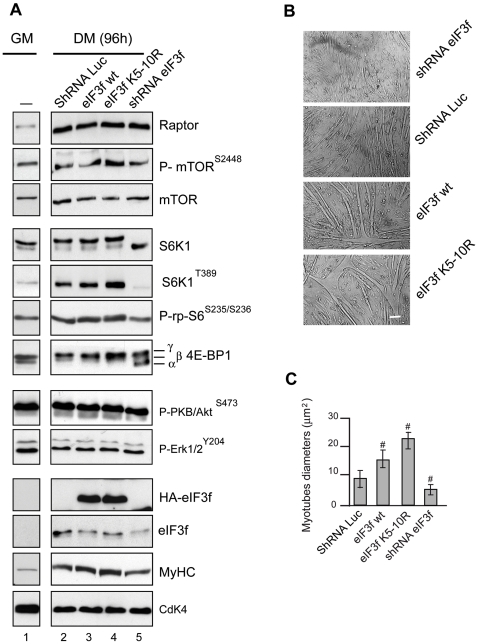
eIF3f regulates the mTORC1 pathway in differentiated myotubes. (**A**) Mouse primary cultured satellite cells were transfected with expression vectors encoding HA-tagged eIF3f wt or the mutant K_5–10_R, or subjected to RNAi-mediated silencing of eIF3f. At 24h posttransfection cells were induced to differentiate. Total lysates from proliferating control cells (GM) or 4 days (DM 96h) differentiated myotubes were analyzed by immunoblotting using the indicated antibodies and phospho-specific antibodies. (**B**) Effects of the overexpression and/or the knockdown of eIF3f on myotubes size. Mouse primary cultured satellite cells were transfected with expression vectors as indicated in (A). Cells were cultured in differentiation medium for 4 days. Bright-field images of differentiated myotubes are shown. Scale bar, 20 µm. (**C**) Myotube mean diameter of experiments as in (A) was measured. Data represent the average ± s.e.m for three experiments ^#^, *P*<0,05 compared to control. At least 150 myotubes for each condition were analyzed.

To examine the role of eIF3f regarding the mTORC1 activity, we transfected expression vectors coding for HA-tagged eIF3-f wt, the mutant eIF3f K_5–10_R or a shRNAi against eIF3f in mouse primary muscle cells and then we examined during terminal muscle differentiation the components of the Akt/mTORC1 pathway known to play a prominent role in muscle hypertrophy [15]. As shown in Figure 3A, mTOR and raptor expression was up regulated during muscle differentiation [34] independently of eIF3f expression while S6K1 activity increased and 4E-BP1 phosphorylation remained elevated. As expected, overexpression of eIF3f wt was characterized by up-regulation of the myosin heavy chain and increased myotube diameters. This phenomenon was associated with increased hyperphosphorylated forms of S6K1 and 4E-BP1. Higher phosphorylation of S6K1 in Thr389 and rpS6 in Ser235/236 were also observed (Figure 3A, lane 3, Figures 3B and 3C). Furthermore, overexpression of the mutant eIF3f K5-10R lead to a hypertrophic phenotype and the phosphorylation of these downstream targets of mTORC1 were even higher than those observed with eIF3f wt (Figure 3A lane 4, Figures 3B and 3C). In addition, rapamycin treatment destabilized the mTOR-raptor complex [10] but does not completely abolished eIF3f effects (Figure S1). Altogether, these data show that the eIF3f-mediated hypertrophy is characterized by increased activity of mTORC1.

To further address the role of eIF3f in mediating the mTOR signaling, primary skeletal myotubes were subjected to shRNAi-mediated silencing of eIF3f. When myoblasts expressing the shRNA were induced to differentiate, the eIF3f-knockdown cells presented a defect in the muscle differentiation process, with a significant reduction of the fibers diameter and the expression of the MyHC (Figure 3A lane 5, Figures 3B and 3C). Moreover, eIF3f knockdown resulted in significant decreases in the phosphorylation of S6K1 in T389 and its target the rpS6 in Ser235/236, accompanied with increasing non-phosphorylated forms of 4E-BP1. Furthermore, specific repression of eIF3f did not induce significant changes in either the levels or the phosphorylation of the mitogen-activated protein kinases Erk1 and Erk2 and PKB/Akt (Figure 3A). Thus, we conclude that eIF3f exerts its myogenic function by controlling the mTORC1/S6K1 pathway during muscle terminal differentiation.

### Identification of a Conserved TOS Motif in the C-Terminus of eIF3f That Is Essential for mTOR-Raptor Activation

**Figure 4:**
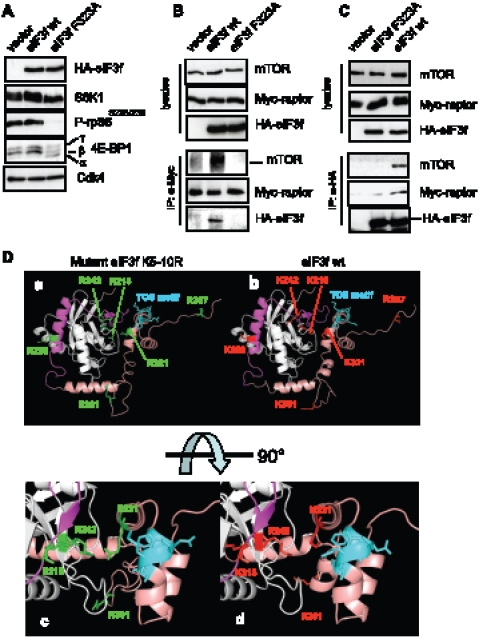
A TOS motif in eIF3f is necessary for binding to mTOR-raptor. (**A**) Effects of TOS mutation on the activity of mTORC1. Mouse primary muscle cells were transfected with expression vectors encoding HA-tagged eIF3f wt, HA-tagged TOS motif mutant eIF3f F323A and the empty vector. At 24h post-transfection cells were induced to differentiate. Protein expression and phosphorylation levels were assayed from lysates of 4 days differentiated myotubes by immunoblotting. (**B**) Mouse primary muscle cells were co-transfected with expresion vector encoding Myc-raptor and either HA-eIF3f wt, the TOS motif mutant HA-eIF3f F323A and/or the empty vector. Transfected cells were induced to differentiate during 4 days and lysed in immunoprecipitation buffer without detergent. Total cellular extracts were subjected to immunoprecipitation with anti-Myc antibody, followed by immnoblotting. Asterisk indicates a non specific band . (**C**) Mouse primary muscle cells were transfected, differentiated and lysed as indicated in ***B***. Total cellular extracts were subjected to immunoprecipitation with anti-HA antibody, followed byimmunoblotting. (**D**) Modeling of eIF3f shows that the functional TOS is accessible for eIF3f/raptor interaction. Models of the three-dimensional structures of wild type and mutant eIF3f K5-10R. The left-side images correspond to mutant eIF3f K_5–10_R (panels a and c), right-side to eIF3f wt (panels b and d). The mutated lysines are coloured red, and corresponding arginines are colored green. The C-terminal arm is colored salmon. The mTOR binding region is colored pink. The TOS motif is colored blue. The bottom panel is a zoom on the central region in a 90° rotation.

The mTOR-raptor interaction has been shown to involve multiple subdomains in raptor protein and a large region of mTOR suggesting that the two proteins make extensive contact between each other [10]. Raptor association with mTOR is required for efficient S6K1 and 4E-BP1 phosphorylation and raptor has been suggested to function as a scaffolding protein that brings mTOR in close proximity to its substrates [36]. We focused our work on defining mTOR-raptor interactions with the eIF3 subunit, eIF3f. Deletion of the C-terminus of eIF3f has been shown to repress mTOR activity in muscle cells (Figure 2D). Because recent reports demonstrated that TOS (TOR Signaling) motif is necessary for the binding of S6K1 and 4E-BP1 to raptor [37], [38], this prompted us to research whether such a motif is present in the C-terminal part of eIF3f. We found a five amino acid sequence in eIF3f, FETML (amino acids 323–327) that is evolutionary conserved and matched with other TOS motif that have been shown to function in their respective proteins (Table 1). To determine the importance of this motif for mTOR-raptor function and because the first position in the TOS motif is critical, we mutated the phenylalanine residue to alanine (F323A). Then, we investigated whether the TOS motif is required for mTORC1 regulation by eIF3f. We cotransfected HA-tagged eIF3f wt and/or the TOS motif mutant eIF3f (F323A) with Myc-tagged raptor in mouse primary muscle cells. The introduced mutation had a dramatic inhibitory effect on mTORC1 activity (Figure 4A) and lead to a lack of muscle differentiation (Figure S2). In addition, the TOS motif mutant eIF3f (F323A) failed to coimmunoprecipitate with raptor and mTOR whereas the eIF3f wt coimmunoprecipitated (Figures 4B and 4C) indicating that the TOS motif in eIF3f is required both for the binding and the activity of mTOR-raptor in muscle cells.

**Table 1:** TOS motifs that have been shown to function in their respective proteins.

Sequence		eIF3 proteins	
S6K1	FDIDL	Human	FETML
S6K2	FDIDL	chimpanzee	FETML
4E-BP1	FEMDI	cow	FETML
4E-BP2	FEMDI	rat	FETML
4E-PB3	FEMDI	mouse	FETML
HIF1α	FVMVL	chicken	FETML
PRAS40	FVMDE	zebra fish	FENML
eIF3f	FETML		

### Mutation of the C-Terminal Lysines in eIF3f Increases Interaction with mTOR and Raptor

Our previous results demonstrated that the mutant eIF3f K_5–10_R leads to increased hypertrophy when compared to the wild-type protein *in vivo* and *in cellulo* [29]. These observations agree with the data presented in Figure 3, overexpression of the eIF3f mutant K_5–10_R is correlated with increased activation of downstream targets of mTORC1, but exactly how this mutant protein mediates this enhanced activity remains unknown. To investigate how the mutation of C-terminal lysines affected mTORC1 activity, computational modeling of eIF3f was first undertaken. The sequence of mouse eIF3f can be divided in three sub-domains. The N-terminal domain (1–86) is predicted unfolded. The central region (87–260) is a Mov34 domain. The C-terminal region (261–350) is folded, and not found in other proteins containing a Mov34 domain. The three-dimensional structure of central and C-terminal regions was modeled using two support structures, one for each region. For the central region, the MPN domain of the 26S proteasome non-ATPase regulatory subunit 7 (PDB code 2O95; Ref 39) was used as support structure. Among the proteins with known three-dimensional structure containing a Mov34 domain, it is the one sharing the highest sequence identity with eIF3f (29%); it is also the longest one. In particular, it contains, as compared to other Mov34 domain structures, a supplementary C-terminal alpha helix that is also present in eIF3f.

For the C-terminal region, possible support structures were searched for using the @tome server [40]. Among the possible support structures with comparable scores, we have chosen the C-terminal sub-domain of a putative ribose 5-phosphate isomerase from Novosphingobium aromaticivorans (PDB code 3C5Y, unpublished). This choice was based on two elements: firstly, although the sequence identity is very low (12.1%), conserved positions in eIF3f correspond to conserved positions in 3C5Y; secondly, the concerned C-terminal region in 3C5Y is not shared by most proteins homologous to 3C5Y, showing that this region might be an autonomous domain. These two elements could not be established for any of the other candidates. In order to model the two regions together, the two support structures were assembled in a chimerical structure. Prior to this, the 2O95 structure was minimized; using rigid body, then simulated annealing, to bring the C-terminal helix against the structure. Indeed, in the dimmer, this helix rotates outwards to make extensive contacts with the second monomer. To orient the two domains relative to each other, we took advantage of the fact that the helix separating the two domains can be modeled using as support structure either the C-terminal helix of 2O95, or the first helix of the 3CY5 C-terminal domain. Thus, these two helices were superimposed. Noteworthy, both in the chimerical structure and in 3CY5, 3CY5 C-terminal domain appears as an “arm” closing up on the rest of the structure.

The wild type and mutants were modeled separately. Both models were evaluated, giving good PROSA scores (−4.88 for wt and −5.25 for mutant). In both cases, Verify3D also shows good score for most of the model except for regions where the scores are very low or negatives. The first region corresponds to a region in 2O95, which also exhibits negative scores. The second and third regions correspond to loops that have no equivalent in the support structures. The last region corresponds to the C-terminal amino acids that have no equivalent in 3CY5. Noteworthy, the “arm”, modeled from that of 3CY5 allows covering hydrophobic regions that would otherwise be exposed (Figure 4D). In both models (mutant and wild-type), the TOS motif is exposed. Three of the positions mutated in eIF3f K_5–10_R appear very close to this TOS motif and could explain the differences observed in affinity of eIF3f for mTOR/raptor.

**Figure 5:**
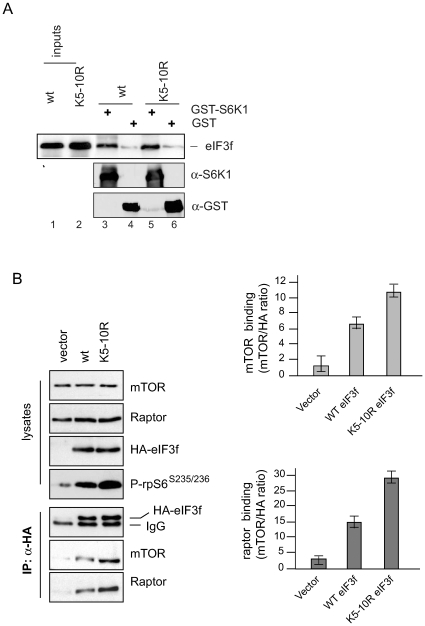
The hyperactive eIF3f mutant K_5–10_R shows increased affinity for binding to mTOR/raptor. (**A**) Interaction of S6K1 with eIF3f wt and mutant K_5–10_R *in vitro*. GST or GST-S6K1 beads were incubated with *in vitro* translated HA-tagged eIF3f wt or mutant K_5–10_R. Bound proteins were eluted, subjected to SDS-PAGE and analyzed by immunoblotting. (**B**) Co-immunoprecipitation of endogenous mTOR/raptor with eIF3f wt and mutant eIF3f K_5–10_R. Mouse primary skeletal muscle cells were transfected with expression vectors encoding HA-tagged eIF3f wt or the mutant K_5–10_R. Cell extracts of 3 days differentiated myotubes were subjected to immunoprecipitation with anti-HA antibody. Immune complexes were subjected to SDS-PAGE and Western blotting. The bar graphs show the ratio of mTOR and raptor recovered relative to HA-tagged eIF3f wt or mutant K_5–10_R. Data represent the combined results from three different experiments.

Since eIF3f serves as a connecting platform between mTOR-raptor and S6K1 in muscle cells (Figure 2) and mutation of the C-terminal lysines in eIF3f increases phosphorylation of S6K1, we assessed the binding affinities of both eIF3f wt and mutant eIF3f K_5–10_R proteins with S6K1 and mTOR-raptor. As previously shown in Figure 2B, the non-phosphorylated form of S6K1 interacts with eIF3f. Thus, we determined whether eIF3f wt and/or the mutant eIF3f K_5–10_R could be captured with the same affinity *in vitro* by using a GST-S6K1 (unphosphorylated) bound to GSH-agarose. As seen in Figure 5A, the amount of *in vitro*-translated eIF3f wt recovered with the S6K1 affinity resin was equivalent to the retained amount of eIF3f mutant K_5–10_R. The same results were obtained by co-immunoprecipitation of HA-tagged eIF3f wt and/or the mutant eIF3f K_5–10_R protein with endogenous S6K1 in rapamycin-treated mouse primary myotubes (data not shown). In contrast, HA-tagged eIF3f mutant K_5–10_R overexpressed in mouse primary myotubes at 4^th^ day of differentiation was able to bind both endogenous mTOR and raptor by about 2-fold higher than HA-tagged eIF3f wt leading to a higher phosphorylation and activity of S6K1 as evidenced by an increased phosphorylation of rpS6 on Ser235/236 residues (Figure 5B). These findings strengthen the notion that the modifications of C-terminal lysines in arginine increase affinity of mTOR-raptor for the TOS motif in the mutant eIF3f K_5–10_R.

### eIF3f Regulates the Association of Translational Components with the 7-Methylguanosine-Cap Complex in Muscle Cells

Our data suggest that during terminal differentiation and hypertrophy eIF3f plays a scaffolding role for the activation of 4E-BP1 and S6K1 by mTORC1. While the precise role of S6K1 in translational control is still poorly understood, it is known that the hypophophorylated 4E-BP1 acts as negative regulator of the cap-binding protein eIF4E. Phosphorylation of 4E-BP1 by mTORC1 promotes its dissociation from the eIF4E bound to the mRNA 7-methylguanosine cap structure, allowing for the recruitment of eIF4G and eIF4A, 40S ribosomal subunits and the ternary complex (eIF2/Met-tRNA/GTP), resulting in the assembly of the preinitiation complex (PIC) [9].

**Figure 6:**
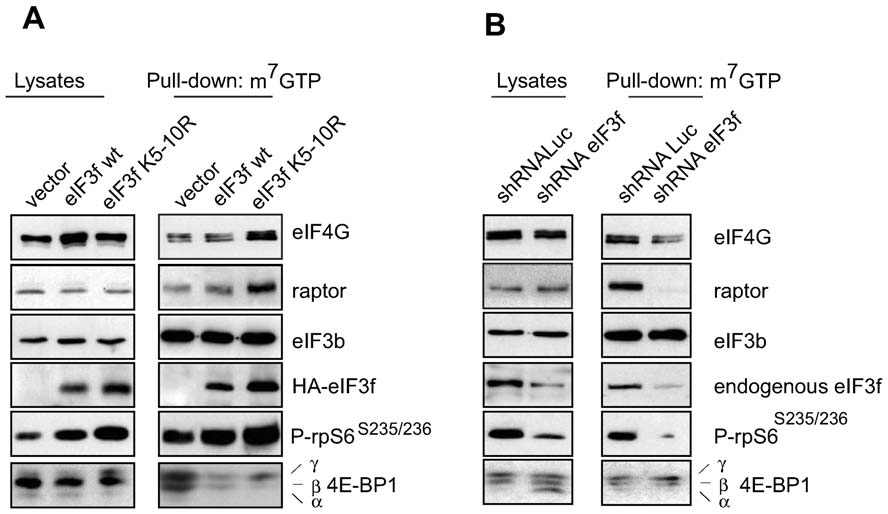
eIF3f regulates the recruitment of translational proteins to the mRNA 7-methylguanosine cap structure. (**A**) Binding of translational components to the 7-methylguanosine cap complex is enhanced by eIF3f. Mouse primary cultured satellite cells were transfected with expression vectors encoding HA-tagged eIF3f wt and/or the mutant eIF3f K_5–10_R and differentiated for 3 days. Cap-binding proteins in lysates were purified by 7-methyl GTP (m^7^GTP) affinity beads. Levels of proteins and phosphoproteins were analyzed by Western blotting. (**B**) eIF3f silencing leads to decreased recruitment of translational components to the m^7^-GTP cap complex. Mouse primary cultured satellite cells were subjected to RNAi-mediated silencing of eIF3f using specific small hairpin RNA. A nonspecific shRNAi was used as control. Cell lysates were purified by m7-GTP and analyzed as described in (A).

We set out to determine the functional consequences of the eIF3f-induced activation of mTORC1 and phosphorylation of S6K1 and 4E-BP1 (Figure 3), thus we investigated the formation of the translational PIC by using a cap-binding assay. Mouse primary muscle cells were transfected with expression vectors coding for HA-tagged eIF3f wt, the mutant eIF3f K_5–10_R or subjected to shRNA-mediated silencing of eIF3f. After 4 days of differentiation, cell extracts were prepared without detergent, incubated with 7-methyl-GTP (m^7^-GTP) Sepharose beads, and the bound proteins were analyzed by immunoblot. HA-eIF3f K_5–10_R showed a higher affinity to copurify on m7-GTP beads when compared to HA-eIF3f wt. We also observed that endogenous eIF4G, raptor and rpS6 were robustly recruited to the m^7^-GTP cap complex and 4E-BP1 was released in myotubes overexpressing eIF3f wt when compared to empty vector, and even higher in those overexpressing the mutant eIF3f K_5–10_R (Figure. 6A). In contrast, eIF3f knockdown abolished the recruitment of these translational components to the m^7^-GTP cap structure and increased the retention of 4E-BP1, when compared to shRNA Luc (Figure 6B). These results show that eIF3f is involved in the proper assembly of the translation initiation complex in skeletal muscle cells.

### eIF3f Activates the Cap-Dependent Translation in Muscle Cells

**Figure 7:**
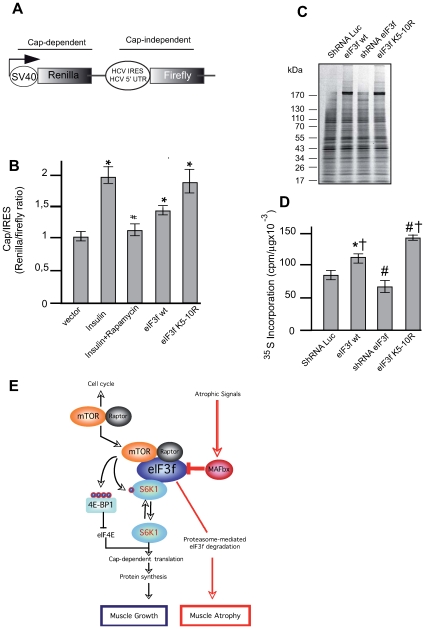
eIF3f regulates cap-dependent translation. (**A**) Structure of the bicistronic reporter plasmid allowing cap-dependent expression of renilla luciferase and expression of firefly luciferase dependent on HCV IRES. (**B**) Overexpression of eIF3f modulates cap-dependent translation. Mouse primary cultured satellite cells were cotransfected with the bicistronic reporter vector and expression vectors encoding HA-tagged eIF3f wt and the mutant eIF3f K_5–10_R. Twenty-four hours posttransfection cells were grown for an additional 24h in 20% serum (control), stimulated with insulin or pretreated with rapamycin and stimulated with insulin for and additional 24 h. Cells transfected to express eIF3f wt or the mutant eIF3f K_5–10_R were grown in 20% serum. Luciferase activities were measured by a dual-luciferase assay. The ratio of Renilla (Cap-dependent) to Firefly (IRES-dependent) luciferase activity was calculated. Data are presented as the mean ± standard error from three independent experiments carried out in triplicate, *P<0,05 compared to control; ^#^ *P*<0,05 compared to Insulin + rapamycin. (**C**) Mouse primary cultured satellite cells were transfected with expression vectors as described in (B) and/or subjected to shRNAi-mediated silencing of eIF3f prior to labeling new protein synthesis with ^35^S methionine. Newly synthesized proteins were separated by SDS-PAGE, and visualized by autoradiography. (**D**) Newly synthesized proteins from three experiments as in (C) were quantified. *P = 0,002 and ^#^ *P*<0,001 compared to control; ^†^ *P*>0,001 compared to shRNAi eIF3f. (**E**)**.** Model depicting the central role of eIF3f in the signaling pathways controlling skeletal muscle mass.

Translation initiation is the rate-limiting step in cap-dependent protein translation and the majority of protein synthesis is though to be cap-dependent [41]. Thus, we set out to determine whether eIF3f contribute *in vivo* to cap-dependent translation. For this, we used a dual luciferase reporter system previously described (Figure 7A) [27], [42]. Using this assay, we first measured the effect of insulin known to induce hypertrophy and rapamycin (Figure S3) on cap-dependent translation in muscle cells. As shown in Figure 7B, insulin induced a 2-fold increase in cap-dependent over cap-independent translation rates in muscle cells. This effect was completely rapamycin sensitive suggesting that signaling through the mTOR pathway modulated the insulin-induced cap-dependent translation. Then, we measured the effect of eIF3f on cap-dependent translation. As shown in Figure 7B, eIF3f wt overexpression led to increase in translation rates in muscle cells. Interestingly, overexpression of the mutant eIF3f K_5–10_R increased the cap-dependent translation in the same order as observed with insulin. Consistently with this, we observed that overexpression of eIF3f in primary skeletal myotubes increased total protein synthesis by about 23% for the wt protein and 60% for the mutant K_5–10_R, when compared with empty vector. In contrast, knockdown of eIF3f in myotubes reduced global protein synthesis by about 25% (Figures 7C and 7D).

## Discussion

The inhibitory effects of rapamycin on skeletal muscle hypertrophy and the critical role of raptor for muscle function and prolonged survival [19] suggest the involvement of mTORC1 pathway in the muscle regulation and hypertrophy. The downstream effector of mTOR, S6K1 has also been shown to be a regulator in this process [18]. In the present work, we provided several important new insights concerning the implication of the regulatory subunit eIF3f in the control of mTORC1 activation and its implication in the regulation of the Cap-dependent translation in muscle fiber size.

S6K1 and mTOR-raptor physically interact with two different domains of eIF3f. The binding of S6K1 to the eIF3 complex is not mediated by the TOS motif [27]. The Mov34 domain of eIF3f is able to associate with the hypophosphorylated form of S6K1 adds to the evidence of a physical association of eIF3f and S6K1 prior to S6K1 activation. Interestingly, the Mov34 motif in eIF3f interacts directly with the Leucine Charged Domain (LCD) of MAFbx during atrophy [25]. MAFbx could control cell growth by interacting with eIF3f for further degradation by the 26S proteasome, preventing the activation of S6K1 by mTOR. Muscles undergoing atrophy accumulates the inactive form of S6K1 suggesting that degradation of eIF3f mediated by MAFbx participates to S6K1 inactivation during atrophy and raises the question whether MAFbx interacts with free eIF3f molecules or bound to hypophosphorylated S6K1. Indeed association of eIF3f with mTOR and its activation is correlated with S6K1 activation and its respective dissociation from eIF3f. The raptor-mTOR binding to eIF3f physically does displace S6K1 from eIF3f but S6K1 phosphorylation by mTORC1 alters the ability of eIF3f and S6K1 to interact.

In myotubes, the mTOR-raptor activation seems to be dependent of interactions with a TOS motif located in the C-terminal domain of eIF3f. Only eIF3f with an intact TOS motif coimmunoprecipitates with mTOR-raptor (Figures 4B and 4C) and activates mTOR-raptor/S6K1 pathway (Figure 4A). The TOS motif is found in substrates of the mTOR kinase. TOS interacts with the WD40-containing adaptor protein raptor that is required to bring mTOR together with its substrates. eIF3f has been shown to be phosphorylated by CDK11 during apoptosis [43]. Although our preliminary data did not show major posttranslational modifications of eIF3f during muscle differentiation, it is possible that mTOR directly phosphorylates eIF3f and we are currently pursuing this line of investigation to see whether there is any mTOR-dependent and rapamycin-sensitive phosphorylation sites within eIF3f. This TOS motif is derived from the conservation observed in the 4E-BP translation initiation factors and the S6K1, S6K2 kinases and shows a strong beta amphipathicity. The motif alternates between hydrophobic and negatively charged residues. Although the structure of a TOS-Raptor complex is not yet known, the conservation might imply that the motif is bound in a sandwiched pocket. In our model, the TOS motif is exposed, and thus accessible to raptor (Figure 4D). Moreover, this motif is very close to the mTOR binding region. Three of the K/R mutations (positions 218, 242 and 321) lie between these two motifs, and are exposed, thus belonging either to the raptor or to the mTOR binding sites. Consequently these three mutations alone could explain the observed enhancement of the affinity of the mutant for raptor/mTOR (Figure 5). Together with a fourth mutation (position 301), they could also be responsible of a change in the stability of the arm's position (in salmon) relative to the rest of the structure, especially the mTOR-binding region. Mutation at position 258 is situated in the mTOR binding region, and could thus influence the affinity for mTOR. Finally, although the position of the last mutation (position 357) cannot be determined on the model, since the support structure is slightly shorter than eIF3f, it is very likely that this position is in the vicinity of the TOS motif, and could thus belong to the raptor-binding region.

eIF3f is one of two eIF3 subunits that contain a Mov34 motif. The function of this domain is unclear, but it is found in the N-terminus of the proteasome regulatory subunits, eukaryotic initiation factor 3 (eIF3) subunits f and h and in certain subunits in the COP9 signalosome and the lid of the 19S proteasome [20]. The role of eIF3f within the eIF3 complex has not been defined. eIF3f is not found in S. Cerevisiae. However in Schizosaccharomyces pombe eIF3f is essential for viability and depleting eIF3f remarkably decreases global protein synthesis in fission yeast [21]. eIF3f overexpression has been associated with inhibition of HIV-1 replication [22] and with activation of apoptosis in melanoma and pancreatic cancer cells [23]. Changes in the composition of eIF3 represent another potential mechanism for controlling eIF3 function. It has been shown that the amount of eIF3j in eIF3 complex influences the amount of 40S subunit associated with eIF3 [44]. During terminal muscle differentiation the amount of eIF3f increase [25] as well as mTOR and raptor (Figure 3), leading to an increase in S6K1 activation and phosphorylation of rpS6 and 4E-BP1. Recent reports on the biological function of eIF3f in translation and apoptosis in tumor cells demonstrated that eIF3f is down regulated in most human tumors and that overexpression of eIF3f inhibited cell proliferation suggesting a function associated with differentiation [23]. Overexpression of eIF3f in myotubes (Figure 3) and in mouse skeletal muscle [2] induces a massive hypertrophy. mTOR is believed to be a master regulator of skeletal myogenesis by controlling multiple processes through different mechanisms. In particular the formation of mature myotubes requires mTOR kinase activity [45] and mTOR function in skeletal muscle requires only mTORC1 activation [19]. In contrast, eIF3f knockdown was sufficient to induce the repression of S6K1 activity and the lack of myogenic differentiation (Figure 1). Ablation of eIF3f in muscle cells prevents mTORC1 activity, phosphorylation of S6K1, rpS6 and 4E-PB1. This mTOR-signaling pathway has been shown to directly control protein synthesis. Thus increased mTORC1 signaling leads to increased translation. Impaired efficacy of protein synthesis in muscle atrophy via degradation of eIF3f extends previous data in which mTOR inhibition by rapamycin was shown to prevent compensatory hypertrophy and recovery from atrophy [15]. Our data are also consistent with the findings that skeletal muscles of S6K1-deficient mice are atrophic [18]. The targeting of eIF3f in the atrophic pathway regulated by MAFbx in muscle atrophy suggested an unexpected implication for eIF3f in the control of muscle cell size. We have demonstrated that the regulatory eIF3f subunit acts as a scaffold for mTORC1-and S6K1-mediated assembly of the translation initiation complex during muscle terminal differentiation. Our results add to the evidence that both physical and functional links exist between mTOR-raptor, S6K1 and eIF3f. Genetic repression of eIF3f in differentiated skeletal muscle is sufficient to induce atrophy [25] and degradation of eIF3f by MAFbx suppresses S6K1 activation by mTOR. Moreover inhibition of eIF3f degradation (mutant eIF3f K_5–10_R) in MAFbx-induced atrophy maintained S6K1 activation by mTOR (Figure 1) and electroporation of eIF3f K_5–10_R expression vector in mice not only protects against muscle atrophy but also induces hypertrophy [29]. Altogether these observations pinpoint the important role of eIF3f in S6K1 activation and function in the control of muscle mass and size. We recently suggested that eIF3f may act as a « translational enhancer » driving specific mRNAs to polysomes and thus increasing the efficiency of protein synthesis. The role of these proteins in muscle hypertrophy are under investigation. These different observations lead us envision the involment of eIF3f in the regulation of S6K1 and mTOR activation in the assembling of a preinitiation complex specific to mRNA encoding proteins involved in terminal muscle differentiation and hypertrophy. The role of eIF3f as a central element of both atrophy and hypertrophy pathways represents an attractive therapeutic target against muscle wasting. Efficient design of MAFbx specific inhibitors should aim to disrupt its specific interaction with eIF3f by developping compounds to prevent and/or slow down skeletal muscle atrophy.

## Materials and Methods

### Ethics Statement

All animals were handled in strict accordance with good animal practice as defined by the relevant national and/or local animal welfare bodies, and all animal work was approved by the Ministere de l”Enseignement Superieur et de le Recherche (decret N. 4962 du 12 /06/ 2008).

### Reagents

Insulin was purchased from SIGMA. Rapamycin was a kind gift from A. Sotiropoulos (Institut Cochin, Paris, France). The ^35^S-translabel was obtained from Perkin Elmer.

### Plasmid Constructs

Reporter plasmid pRL-HCV-FL was provided by John Blenis (Harvard Medical School, Boston, USA). The coding sequence of human eIF3f obtained from the two-hybrid screen [25] was transferred in pCMV-Myc (Clontech) after Bgl2 restriction digest. The full-length coding sequence and eIF3f mutants were amplified by PCR to introduce BamH1 and EcoR1 sites on each side of the open reading frame and cloned as BamH1 and EcoR1 fragments into pGEX-3X. The coding sequence of mouse eIF3f was amplified by RT-PCR by using forward primer 5′-GGATCCATGGCTTCTCCGGCCGTACCGG-3′ and reverse primer 5′- GAATTCTCAGCCCTGTGGTGAAAACCTC-3′ and subcloned into pCDNA3-T7-3HA after BamH1 and EcoR1 restriction digest. The shRNAi for eIF3f were provided by C. Brou (Institut Paster, Paris). The muscle specific expression plasmid for eIF3f was carrying out first by using the 1256 bp HindIII-BstEII filled-in fragment of the muscle regulatory elements of the Muscle Creatine Kinase (MCK) and subcloned in pEGFP-C1 instead of the pCMV promoter (pMCK-GFP). Then deletion of the GFP sequence was introduced by NheI-BspEI filled-in digestion of pMCK-GFP plasmid and reannealing of the resulting plasmid (pMCK). The HA-tagged eIF3f coding sequence was cloned in sense into SmaI-XbaI sites of the pMCK. The eIF3f deletion mutants were previously described [29]. The eIF3f mutant F323A in which Phe-323 was substituted with Ala was obtained by oligonucleotide-directed mutagenesis as described in the manufacturer's protocole (PCR-based mutagenesis kit, Stratagene). The mutation was confirmed by sequencing. The pRK5/myc-raptor construct has been described [10].

### Cell Cultures

Primary cultures were prepared from male mice from our own breeding stocks. All animals were treated in accordance with institutional and national guidelines. Briefly, mice satellite cells were isolated from the whole muscles of the paw. Cells were plated at a density of 2×10^4^ cell/cm^2^ on Matrigel-coated Petri dishes (BD Biosciences), in 80% Ham's-F10 medium containing glutamine, penicillin and amphotericin B (Invitrogen), supplemented with 20% horse serum. After two days, cells were washed with Ham's-F10 and placed in complete medium supplemented with 5 ng/mL basic fibroblast growth factor. Primary cultures of satellite cells were transfected with 2 µg of total plasmid using Dreamfect (OZBiosciences). High-level transfection efficiency for eIF3f knockdown by shRNA in primary satellite cells was achieved by using a modified protocol for Lipofectamin 2000. Four pShRNAi mouse eIF3f were constructed by inserting at the BamHI-HindIII sites of the pTER+ plasmid [25] a double synthetic oligonucleotides (sense oligo 1: (5′-GATCCCCCGATGAAGTGGCTGTTATTTTCAAGAATAACAGCCACTTCTTCTTTTTGGAAA-3′; antisense oligo 1: 5′-AGCTTTTCCAAAAA GATGAAGTGGCTGTTTACTCTTGAAATAACAGCCACTTCTACGGG-3′. Sense oligo 2: 5′-GATCCCCGCCTATGTCAGCACTTTAATTTTCAAGAATTAAAGTGCTGACATAGGCTTTTTGGAAA-3′; antisense oligo 2: 5′-AGCTTTTCCAAAAAGCCTATGTCAGCACTTTAAT ACTCTTGAAATTAAAGTGCTGACATAGGGGG-3′. Sense oligo 3: 5′-GATCCCCCGCATCGGAGTTGATCTGATTTTCAAGAATCAGATCAACTCCGATGCGTTTTTGGAAA-3′; antisense oligo 3: 5′-AGCTTTTCCAAAAA CGCATCGGAGTTGATCTGACTCTTGAAATCAGATCAACTCCGATGCGGGG-3′). Sense oligo 4: 5′-GATCCCCGAGTGATTGGACTCTTAAGTTTTCAAGAACTTAAGAGTCCAATCACTCTTTTTGGAAA-3′; antisense oligo 4: 5′-AGCTTTTCCAAAAA GAGTGATTGGACTCTTAAGTCTCTTGAAACTTAAGAGTCCAATCACTCGGG-3′.To design the control construct, two sets of oligonucleotide pair (sense: 5′-GATCCCCGTACGCGGAATACTTCGATTCAAGAGATCGAAGTATTCCGCGTACGTTTTTGGAAA-3′ and antisense 5′-AGCTTTTCCAAAAACGTACGCGGAATACTTCGATCTCTTGAATCGAAGTATTCCGCGTAGGG-3′) directed against the Firefly luciferase were inserted into the BamHI-HindIII sites of the pTER+ plasmid. Protein extraction was performed as described previously [25] and ShRNAi efficiency was tested by Western blot (Figure S4).

For microscopy experiments, primary skeletal muscle myotubes cells at 4^th^ day of differentiation fixed in 3% paraformaldehyde in PBS at pH 7.4 for 30 minutes at room temperature. Bright-field images of myotubes were randomly taken and analyzed by the Axiovision 4.4 Software (Zeiss). The Perfect Image v.5.5 Software (Claravision, France) was used to measure diameters of at least 150 myotubes in a region where myonuclei were absent and the diameter was constant.

### Atrophy Assay

Atrophy was induced in cultured myotubes by switching the medium to PBS (100 mM NaCl, 5 mM KCl, 1.5 mM MgSO_4_, 50 mM NaHCO_3_, 1 mM NaH_2_PO_4_, 2 mM CaCl_2_) during the indicated times.

### Immunoprecipitation and Western Blot

Muscle cells were rinsed in cold PBS and lysed in IP buffer (50 mM Tris pH 7.4, 150 mM NaCl, 10% glycerol, 0.5% NP40, 0.5 mM Na-orthovanadate, 50 mM NaF, 80 µM β-glycerophosphate, 10 mM Na-pyrophosphate, 1 mM DTT, 1 mM EGTA and 1 µg/ml leupeptin, 1 µg/ml pepstatin and 10 µg/ml aprotinin). Lysates were precleaned for 30 min with protein-G beads and immunoprecipitated by using standard procedures. Immunoprecipitated proteins were loaded onto 10% SDS/PAGE gels before electrophoretic transfer onto nitrocellulose membrane. Analyses of the mobility of differently phosphorylated forms of 4E-BP1 and S6K1 were made as described previously [29]. Gel loading was normalized to protein concentration. Western blotting was performed by using an ECL kit (Amersham Biotech.) according to the manufacturer's instructions. Blots were exposed with Amersham Biosciences Hyperfilm ECL (GE Healthcare) films. Signals were quantified by gel scan and with the ImageJ software.

### Antibodies

An anti-MAFbx antibody was generated by injecting rabbits with a GST-MAFbx fusion protein corresponding to aa 1-102 of the human MAFbx protein. Antibodies were affinity purified against an MBP-MAFbx fusion protein. Anti-Troponin T monoclonal (JLT-12), anti-Myosin Heavy Chain monoclonal (My32) and anti-FLAG epitope (M2) antibodies were from Sigma, anti-HA epitope (12CA5) was from Roche Applied Science. Polyclonal anti-S6K1 (C-18), monoclonal anti-Raptor (10E10), anti-phospho Tyr204 ERK1/2 polyclonal, monoclonal anti-Myc (9E10) and anti-Cdk4 were purchased from Santa Cruz. Rabbit polyclonal anti-eIF3f was from Rockland Immunochemicals Inc. Anti-mTOR polyclonal, anti-4E-BP1 polyclonal, anti-phospho-S6 (Ser235/236), anti-phospho-Akt (S473), anti-Rheb and anti-eIF4G were from Cell Signaling Technology. The monoclonal anti-Desmin was purchased from DakoCitomation. The Texas red-conjugated F(ab')_2_ fragments of goat anti-mouse IgG and the FITC-conjugated F(ab')_2_ fragments of goat anti-rabbit IgG were obtained from Jackson Immunoresearch Inc.

### Preparation of Recombinant Proteins and GST (Glutathione-S-Transferase) Pull Down Assay

GST-tagged forms of S6K1 and eIF3-f were made by transforming pGEX-3X-eIF3-f constructs and pGEX2T-S6K1 constructs, respectively, into BL21-(DE3)-Lys bacterial cells (Stratagene). Cells were grown to OD_600_ = 0.5−0.7 and induced with 0.1 mM isopropyl α-D-thiogalactopyranoside (IPTG) at 21°C for 12 h. Cell lysis, and affinity purification with glutathione-agarose beads (Sigma) were done as described previously (46). Fusion proteins were collected on Glutathione-Sepharose 4B (Pharmacia) and then the purity of the GST and GST fusion proteins were analyzed by SDS-PAGE followed by Coomassie brilliant blue staining of the gels. 400 µg of myotubes lysate was diluted in binding buffer (20 mM HEPES pH 7.9, 50 mM KCl, 2.5 mM MgCl2, 10% glycerol, 1 mM DTT and 10 µg/ml leupeptin 10 µg/ml pepstatin and 10 µg/ml aprotinin), and pre-cleaned with Glutathione sepharose beads for 30 min. Then the resulting supernatant was incubated with the beads for 3 hours. Beads were washed four times in NTEN buffer at room temperature and then mixed with 1 volume of 2X SDS loading buffer, and bound proteins were analyzed by SDS-PAGE using standard procedures.

### Molecular Modeling

3D structure of eIF3f was modeled using MODELLER 9v7 [47]. Support structure for modeling of the central region (87–260) was found using Psi-Blast [48]. Support structure for the C-terminal region was found using SP^3^ [49], through the @tome2 server [50]. Structures were minimized using Xplor-NIH [51]. Images were obtained using PyMol [52]. Unfolded regions predictions were made using Prelink [53]. Quality of the models was assessed using PROSA [54] and Verify3D [55].

### Cap Pull-Down Assay

Primary skeletal muscle myotubes were lysed in cap lysis buffer (140 mM KCl, 10mM Tris pH 7,5, 1mM EDTA, 4mM MgCl_2_, 1mM DTT, 1% NP-40, 1 mM sodium orthovanadate, 50 mM β-glycerophosphate, 10 mM NaF and proteases inhibitors). 50 µl of detergent-free cap lysis buffer and 20 µl of pre-washed cap beads (m^7^GTP Sepharose 4B from GE Healthcare Lifesciences) were added to 300 µg of cleared lysate and incubated at 4°C overnight with tumbling. The beads were washed twice with 400 µl of cap wash buffer (cap lysis buffer with 0,5% NP-40 instead of 1%) and twice with 500 µl of ice-cold PBS. The beads were boiled in SDS-PAGE sample buffer and the retained proteins analyzed by Western blot.

### Bicistronic Luciferase Assay

Primary skeletal muscle myotubes were transfected with pRL-HCV-FL reporter plasmid [27], [42] and the indicated DNA. Forty-eight hours post-transfection cells were harvested, and the Renilla and Firefly luciferase activity was measured using the Dual-luciferase kit (Promega). Differences in the ratio of Renilla to Firefly luciferase signals were analyzed for statistical significance by one-way ANOVA with Tukey's post test.

### ^35^S Labeling of New Protein Synthesis

Transfected primary skeletal muscle myotubes at 4^th^ day of differentiation were washed once with DMEM lacking cysteine and methionine (DMEM-noS), and the medium was replaced with DMEM-noS with serum. After incubation for 1 h, 50 µCi of ^35^S (Perkin Elmer) was added to the cells for 4 h. Myotubes were washed once with ice-cold PBS and lysed as described above for western blotting. Following separation by SDS-PAGE and treatment with an amplifier fluorographic reagent (GE Healthcare), ^35^S-labelled proteins were visualized by autoradiography.

### Statistics

All data are expressed as the mean ± S.E. Data were evaluated by one-way analysis of variance followed by Tukey's honestly significant differences test (SigmaSTAT software). A *p* value of <0.05 was considered statistically significant.

## Supporting Information

Figure S1 Rapamycin destabilizes the mTOR-raptor/eIF3f interaction. Mouse primary skeletal muscle cells were transfected with expression vectors encoding HA-tagged eIF3f wt or the mutant K5-10R. Cell extracts of 3 days differentiated myotubes were treated for min with 20nM rapamycin prior to immunoprecipitation with anti-HA antibody. Immune complexes were subjected to SDS-PAGE and probed with anti-mTOR, anti-raptor and anti-HA antibodies.
(3.06 MB EPS)

Figure S2 Mutation of the TOS motif in eIF3f represses muscle differentiation in mouse primary muscle cells. Pools of mock, eIF3-f and mutant TOS F323A eIF3f expressing mouse primary muscle myoblasts were cultured in differentiation medium for 4 days. Bright-field images of differentiated myotubes are shown. Scale bar, 20 µm.
(4.17 MB TIF)

Figure S3 Opposite effects of rapamycin and insulin on the terminal muscle differentiation. Mouse primary cultured satellite myoblasts were induced to differentiate in the absence (control) or in the presence for 2 days of 20mM rapamycin and/or for 2-3 days in the presence of 100nM insulin. Cell lysates were prepared and analyzed by immunoblotting.
(5.38 MB EPS)

Figure S4 Specific down regulation of eIF3f expression by ShRNAi. The small interfering RNA (ShRNAi) studies used oligonucleotide complementary RNA with symmetrical two nucleotide overhangs which were cloned in pTer+ and transfected in muscle cells. Twenty hours after transfection, mouse primary myoblasts were induced to differentiation for three days and then totall cellular lysates were analyzed by Western blot with anti eIF3f and anti Cdk4 antibodies respectively. Asterisk indicates a non-specific band.
(1.81 MB EPS)
