# Supplementary figures and images for: The Translation Regulatory Subunit eIF3f Controls the Kinase-Dependent mTOR Signaling Required for Muscle Differentiation and Hypertrophy in Mouse
Source: PLoS One. 2010 Feb 1;5(2):e8994. doi: 10.1371/journal.pone.0008994 (PMC2813880; doi:10.1371/journal.pone.0008994)

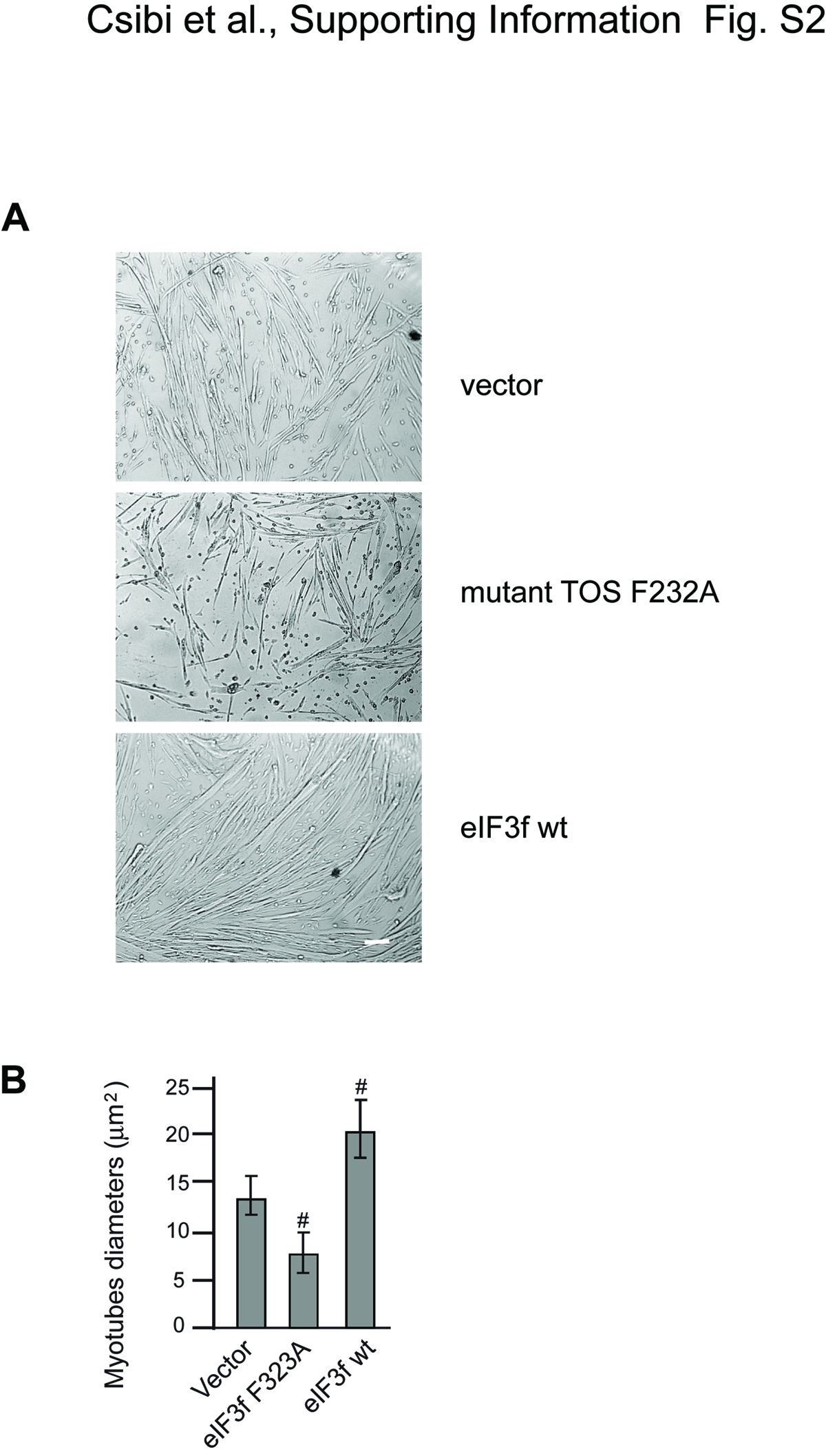

Supplement: Figure S2 — Mutation of the TOS motif in eIF3f represses muscle differentiation in mouse primary muscle cells. Pools of mock, eIF3-f and mutant TOS F323A eIF3f expressing mouse primary muscle myoblasts were cultured in differentiation medium for 4 days. Bright-field images of differentiated myotubes are shown. Scale bar, 20 µm. (4.17 MB TIF) [file pone.0008994.s002.tif]
